# Immunological Response to Single Pathogen Challenge with Agents of the Bovine Respiratory Disease Complex: An RNA-Sequence Analysis of the Bronchial Lymph Node Transcriptome

**DOI:** 10.1371/journal.pone.0131459

**Published:** 2015-06-29

**Authors:** Polyana C. Tizioto, JaeWoo Kim, Christopher M. Seabury, Robert D. Schnabel, Laurel J. Gershwin, Alison L. Van Eenennaam, Rachel Toaff-Rosenstein, Holly L. Neibergs, Bovine Respiratory Disease Complex Coordinated Agricultural Project Research Team, Jeremy F. Taylor

**Affiliations:** 1 Embrapa Southeast Livestock, São Carlos, São Paulo, Brazil; 2 Division of Animal Sciences, University of Missouri, Columbia, Missouri, United States of America; 3 Department of Veterinary Pathobiology, College of Veterinary Medicine, Texas A&M University, College Station, Texas, United States of America; 4 Department of Pathology, Microbiology & Immunology, School of Veterinary Medicine, University of California Davis, Davis, California, United States of America; 5 Department of Animal Science, College of Agriculture, University of California Davis, Davis, California, United States of America; 6 Department of Animal Sciences, College of Veterinary Medicine, Washington State University, Pullman, Washington, United States of America; University of Alabama at Birmingham, UNITED STATES

## Abstract

Susceptibility to bovine respiratory disease (BRD) is multi-factorial and is influenced by stress in conjunction with infection by both bacterial and viral pathogens. While vaccination is broadly used in an effort to prevent BRD, it is far from being fully protective and cases diagnosed from a combination of observed clinical signs without any attempt at identifying the causal pathogens are usually treated with antibiotics. Dairy and beef cattle losses from BRD are profound worldwide and genetic studies have now been initiated to elucidate host loci which underlie susceptibility with the objective of enabling molecular breeding to reduce disease prevalence. In this study, we employed RNA sequencing to examine the bronchial lymph node transcriptomes of controls and beef cattle which had individually been experimentally challenged with bovine respiratory syncytial virus, infectious bovine rhinotracheitis, bovine viral diarrhea virus, *Pasteurella multocida*, *Mannheimia haemolytica* or *Mycoplasma bovis* to identify the genes that are involved in the bovine immune response to infection. We found that 142 differentially expressed genes were located in previously described quantitative trait locus regions associated with risk of BRD. Mutations affecting the expression or amino acid composition of these genes may affect disease susceptibility and could be incorporated into molecular breeding programs. Genes involved in innate immunity were generally found to be differentially expressed between the control and pathogen-challenged animals suggesting that variation in these genes may lead to a heritability of susceptibility that is pathogen independent. However, we also found pathogen-specific expression profiles which suggest that host genetic variation for BRD susceptibility is pathogen dependent.

## Introduction

Bovine respiratory disease (BRD) is the most significant health problem faced by feedlots worldwide and is responsible for 70–80% of morbidities and 40–50% of mortalities in the United States [1]. The precise cost of BRD to the industry is not well understood [2], but losses of at least $1 billion annually have been estimated considering costs of prevention, treatment, loss of productivity and labor [3]. Despite BRD being one of the most common and expensive diseases of feedlot cattle, annual morbidity and mortality rates have increased despite the extensive use of vaccines and antimicrobials [4]. BRD is caused by a number of bacterial and viral pathogens which are collectively referred to as the bovine respiratory disease complex (BRDC). This complex includes bovine herpes virus type 1 also known as infectious bovine rhinotracheitis (IBR), bovine parainfluenza type 3 virus (PI3), bovine viral diarrhea virus (BVDV), bovine respiratory syncytial virus (BRSV), and bacterial pathogens *Arcanobacterium pyogenes*, *Mannheimia haemolytica*, *Pasteurella multocida*, *Histophilus somni* and *Mycoplasma bovis* [5]. However, the onset of disease is usually associated with environmental stressors, a susceptible host and the presence of both viral and bacterial pathogens [4].

Stress resulting from management procedures can predispose calves to an initial infection by a viral pathogen which may suppress the animals’ defense mechanisms, facilitating a secondary infection due to the colonization of the lower respiratory tract by bacteria [6]. While respiratory viruses are capable of causing a clinical disease that is consistent with BRD in the absence of a bacterial co-infection [7], viral infection is usually considered to be the precursor to a secondary bacterial infection which leads to the disease syndrome of BRDC [6,8]. Although the importance of many environmental and management stress factors, such as weaning and transportation are well understood, as is the fact that susceptibility to BRD is lowly to moderately heritable [9], the genetic dissection of the specific loci underlying susceptibility has yet to be tackled. Two approaches are commonly used either individually, or in conjunction, for the dissection of loci underlying phenotypic variation. Genome-wide association analysis is used to identify regions of the genome which harbor variants that have large effects on phenotypic variation and gene expression studies are used to identify transcripts which differ in abundance between individuals which differ in phenotype. When these data are integrated, differentially expressed (DE) genes which reside in genomic regions that have been shown to be trait-associated suggests a direct role for these genes in regulating trait variation [10–12].

The identification of bovine genes and networks which influence host biological response to bacterial and viral infections is an important step towards identifying the specific genetic variants within the bovine genome which enable the establishment of infection and reduce the efficacy of immune responses. These variants may be directly selected in livestock populations to reduce the prevalence of multi-agent diseases such as BRDC. The aim of this research was to compare, relative to controls, the bronchial lymph node transcript levels of steers produced from Angus bulls mated to advanced generation Hereford-Angus crossbred cows that were experimentally challenged with IBR, BVDV, BRSV, *M*. *haemolytica*, *P*. *multocida* or *M*. *bovis*. Bronchial lymph nodes were chosen for analysis because the immune response to pathogens in the lung is important to the host response and these nodes contain lymphocytes that come from the lung. Moreover, these nodes are where antigen presentation occurs and where the T and B cells are stimulated. Consequently, bronchial lymph nodes provide the best window into lymphocyte responses within the infected lung compartment.

## Materials and Methods

### Animal ethics statement

This study was specifically approved by the Institutional Animal Care and Use Committee of the University of California at Davis with animal use protocol #16424.

### Animal sampling and challenge

The sampling of the experimental animals and the design and implementation of the challenge experiment are described in a companion paper. Briefly, steers produced by mating Angus sires to advanced generation Angus-Hereford crossbred dams were obtained from the Sierra Field Station of the University of California Davis located in Brown’s Valley, CA. Blood was collected and steers seronegative, or with the lowest titers against each bacterial and viral pathogen, were selected. The six to eight month old steers were transported to the University of California Davis where they were maintained in pens, fed a 65% concentrate starter diet and were provided water *ad libitum*. The challenge studies were performed sequentially starting with the control animals and with animals housed in groups by control or challenge pathogen. Strict biosecurity protocols were employed including a period between experimental challenges to prevent cross-infection.

Two experiments were conducted during the summers of 2011 and 2012, comprising an initial pilot project designed to determine the conditions for optimization of the challenge experiment and the final optimized challenge experiment, respectively. Optimized doses administered to groups of N = 4 animals challenged with one of the following viruses (BRSV, BVDV and IBR) via a nebulizer or bacteria (*M*. *haemolytica*, *P*. *multocida* and *M*. *bovis*) administered by intratracheal tube inserted directly into the mid-trachea were: BRSV (1.6 × 10^5^/ml × 8.5 ml/animal), IBR (1.0 × 10^7^/ml × 8.5 ml/animal), BVDV (2.0 × 10^8^/ml × 8.5 ml/animal); *M*. *haemolytica* (4.8 × 10^11^ CFU/animal), *P*. *multocida* (1.13 × 10^11^ CFU/animal) and *M*. *bovis* (7.0 × 10^10^ CFU/animal). In addition, two control animals were aerosol administered with 8.5 ml of tissue culture media via a nebulizer and two additional control animals were inoculated by intratracheal instillation with 8.5 ml of phosphate buffered saline. The volume of phosphate buffered saline given by intratracheal inoculation was followed by two volumes of air to make sure that no saline was left in the tube; whereas, the tissue culture media was given by aerosol to mimic one mechanism of viral exposure. One animal challenged with *M*. *bovis* was euthanized early due to extreme disease and tissues were not collected, leaving 27 animals with bronchial lymph node tissues available for analysis from the optimized study. Strains were obtained as follows: BRSV—Dr. Laurel Gershwin, University of California, Davis (CA-1); IBR—California Animal Health and Food Safety Laboratory, clinical isolate (ATCC, lot: VR188 LA); BVDV—Dr. Chris Chase, South Dakota State University (890); *M*. *haemolytica* (89020807N) and *P*. *multocida* (232)—Dr. Anthony Confer, Oklahoma State University; *M*. *bovis* (428E)—Dr. Ricardo Rosenbush, Iowa State University.

Clinical scores were recorded daily from 3 days before challenge until euthanasia using a scoring system based on a combination of systems described elsewhere [13–15]. None of the animals were considered to require treatment with analgesics or anesthetics and these were not provided. Steers were necropsied when clinical signs were determined to have peaked but with the exception of the animal challenged with *M*. *bovis* that was euthanized early, these were not considered to be humane end-points. Animals were euthanized on days 7, 15, 6, 5, 15 and 6 post-challenge, when clinical signs had peaked for BRSV, BVDV, IBR, *M*. *haemolytica*, *M*. *bovis* and *P*. *multocida*, respectively. After the steers were euthanized, lungs and other viscera were removed for examination by a certified veterinary pathologist. Larynx, trachea, bronchi, and lungs were examined and lesions were recorded to estimate consolidation levels.

### RNA isolation and sequencing

One milliliter of Trizol Reagent (Invitrogen, Carlsbad, CA) was added to 50–100 mg of frozen bronchial lymph node sample, which was immediately homogenized. Samples were centrifuged at 12,000 g for 10 min at 4°C and 200 **μ**l of chloroform was added after transferring the aqueous layer to a fresh tube. After another centrifugation at 12,000 g for 10 min at 4°C, RNA from the aqueous layer was precipitated first with 500 **μ**l of isopropanol and then washed with 1 mL of 75% ethanol. The pellet was dissolved in 100 **μ**l DEPC water and placed at 4°C overnight. The following day, the RNA sample was DNase1 treated to remove potential contamination from genomic DNA. RNA purity and concentration was established using a NanoDrop 1000 v1.3.2 (Thermo Scientific, Wilmington, DE). The absence of RNA degradation was first assessed by electrophoresis of 1 **μ**g of RNA on a 1.0% agarose gel. Finally, the quality of each RNA sample was evaluated using an Agilent 2100 Bioanalyzer (Agilent Technologies, Santa Clara, CA) with an RNA NanoChip.

Preparation of the mRNA samples for sequencing was performed by Global Biologics (Columbia, MO) using the TruSeq RNA Sample Preparation Kit (Illumina®, San Diego, CA) and 10 **μ**g of total RNA from each bronchial lymph node. Polyadenylated RNA was purified from the total RNA using oligo dT magnetic beads, then fragmented with divalent cations under elevated temperature. First strand cDNA synthesis was accomplished using random hexamer primers followed by second strand synthesis. Double-stranded cDNA underwent end repair and the 3’ ends were adenylated. Finally, universal adapters were ligated to the cDNA fragments and solid phase PCR was performed to produce the sequencing library. Following library construction, each sequencing library was evaluated using an Agilent 2100 Bioanalyzer and equimolar amounts from each library were pooled to create 5 pools which were individually sequenced (2 × 50 bp) on one lane of a HiSeq 2000 instrument with the exception of pool 5 which was sequenced (2 × 50 bp) on two lanes. An error in library pooling resulted in low sequence coverage for one individual for which additional 2 × 100 bp reads were generated. These reads were trimmed to 2 × 50 bp prior to analysis.

The sequence data were submitted to the National Center for Biotechnology Information Sequence Read Archive under accession number SRP052314. S1 Table contains the sample identification for the RNA-Seq data that corresponds to each of the experimentally challenged animals.

### Processing of sequence reads

Sequence reads were filtered for quality and adapter sequences were trimmed using a custom Perl script that identifies exact string matching to a user-supplied adapter sequence as described in [16].

### Read alignment

Computations were performed on the HPC resources at the University of Missouri Bioinformatics Consortium (UMBC). TopHat v2.0.6 [17] was used to align the trimmed reads to the *Bos taurus* virtual transcriptome build and the UMD3.1 reference genome by providing both a transcript file and the reference genome assembly. TopHat first extracted transcript sequences and used Bowtie to align reads to the virtual transcriptome build. The reads that could not be mapped to the virtual transcriptome were next mapped to the genome assembly. These reads were converted to genomic mappings (spliced as necessary) and merged with the novel transcriptome mappings and junctions. A total of 2 mismatches were allowed in alignment.

### Transcript assembly and quantification

Cufflinks v2.0.2 [18] was used to assemble the aligned reads into a parsimonious set of transcripts for each sample individually. Cufflinks treated regions of the genome that were covered by sequence reads as potential exons and used the mapped junctions to assemble transcripts. Cufflinks also estimates transcript abundances as Fragments Per Kilobase of exon per Million fragments mapped (FPKM), which normalizes transcript expression for transcript length and the total number of sequence reads per sample. The reference annotation supplied to Cufflinks was used to guide the reference annotation-based transcript assembly. The output included all reference transcripts as well as any novel assembled genes and isoforms.

### Testing for differential expression

Cufflinks assemblies for all of the samples were merged using Cuffmerge v2.0.2 which also runs Cuffcompare to annotate the merged assembly and filter a number of transfrags that are likely to be artifacts. The available annotation file was provided to enable the merging of assembled contigs into novel and known isoforms and to maximize the overall quality of the assembly. The reference annotation file was used downstream in Cuffdiff to estimate gene expression levels for each exposure group and to test these groups for DE genes. P-values were corrected for multiple testing (q value) using the Benjamini-Hochberg correction [19]. Cuffdiff estimates the FPKM for each transcript, primary transcript, and gene in each sample. The FPKM values for all genes were used to construct a multidimensional scaling (MDS) plot to investigate the relationships between samples and the within- and between-challenge group gene expression profiles. From among the DE genes, the 200 with the largest FPKM variances across all biological replicates were used to construct a heatmap by hierarchical clustering analysis. All data exploration and visualization was performed using the CummeRbund package [18] implemented in the R environment.

### Annotation of differentially expressed genes

The Database for Annotation, Visualization, and Integrated Discovery (DAVID) v6.7 [20] was used to annotate and interpret the DE gene lists produced by Cuffdiff. DAVID software identifies enriched biological themes and gene ontology (GO) terms and clusters functionally related genes and annotation terms for gene lists with EASE scores < 0.1. The Functional Annotation Tool was used to determine the most relevant GO terms within each DE gene list. The Functional Annotation Clustering algorithm was used to generate a report of related annotation terms and groups of annotation clusters. DAVID Pathway was also used to map genes onto pathways enriched for DE genes.

The Ingenuity Pathway Analysis software (IPA; http://www.ingenuity.com) was also used to discover and explore biological processes and the roles of DE genes. The Ingenuity Pathways Knowledge Base synthesizes relationships such as those between genes, mRNAs and proteins and can be used to identify networks and pathways with an overrepresentation of DE genes.

## Results

### Sequencing throughput, read alignment and assembly

To obtain insights into the molecular basis of immune response in Angus × Hereford steers experimentally challenged with bacterial or viral pathogens most commonly associated with BRDC, we first searched for bronchial lymph node transcript abundance variation among the 23 challenge and four control animals. In this process, we discovered that one of the control animals failed to cluster with the other three biological control replicates in the MDS plot (PC1 *vs* PC2) and, consequently, it was excluded from the analysis (data not shown). We generated an average of 49,360,049 (2 × 50 bp) paired-end reads for each sample and the overall read alignment rate achieved by TopHat ranged from 78.7 to 87.5%.

Alignment files from TopHat were provided to Cufflinks to create a transcriptome assembly for each animal and these were then merged across all animals using Cuffmerge. The identification of full-length isoforms from incompletely reconstructed fragments is not generally reliable without experimental confirmation. Moreover, assembly errors occur and accumulate when several assemblies are merged [21]. Because these RNA-seq data were from a relatively well-annotated organism, and the number of potential novel isoforms appeared to be very large (68.33% of the identified transcripts; Table 1), we chose to not use the merged assembly file in the downstream Cuffdiff analysis to avoid the potential for bias caused by assembly errors [21]. The large number of potentially new isoforms was expected because most human multi-exon genes are known to undergo alternative splicing [22]. We manually evaluated 100 potentially new isoforms by randomly sampling transcripts within different size ranges and searching the NCBI ‘nr’ database for these sequences with BLAST and observed that 76% were new isoforms, 8% were likely assembly artifacts, 10% represented incompletely assembled reference genes (i.e., missing exons or possessing larger 3’ or 5’ end sequences) and 6% had retained introns.

**Table 1:** Transcript Classifications Reported by Cuffmerge for the Merged Assemblies.

Cufflinks Class Description	No. of transcripts	%
Complete match of intron chain	25,197	28.45
A transfrag falling entirely within a reference intron	72	0.08
Potentially novel isoform (fragment)	60,526	68.33
Single exon transfrag overlapping a reference exon and at least 10 bp of a reference intron	0	0.00
Generic exonic overlap with a reference transcript	1,323	1.49
Possible polymerase run-on fragment	1	0.00
An intron of the transfrag overlaps a reference intron on the opposite strand	0	0.00
Exonic overlap with reference on the opposite strand	1,294	1.46
Repeat	0	0.00
Multiple classifications	0	0.00
Unknown, intergenic transcript	166	0.19
Total	88,581	100

### Quality control and exploration of relationships between challenge groups

**Fig 1:**
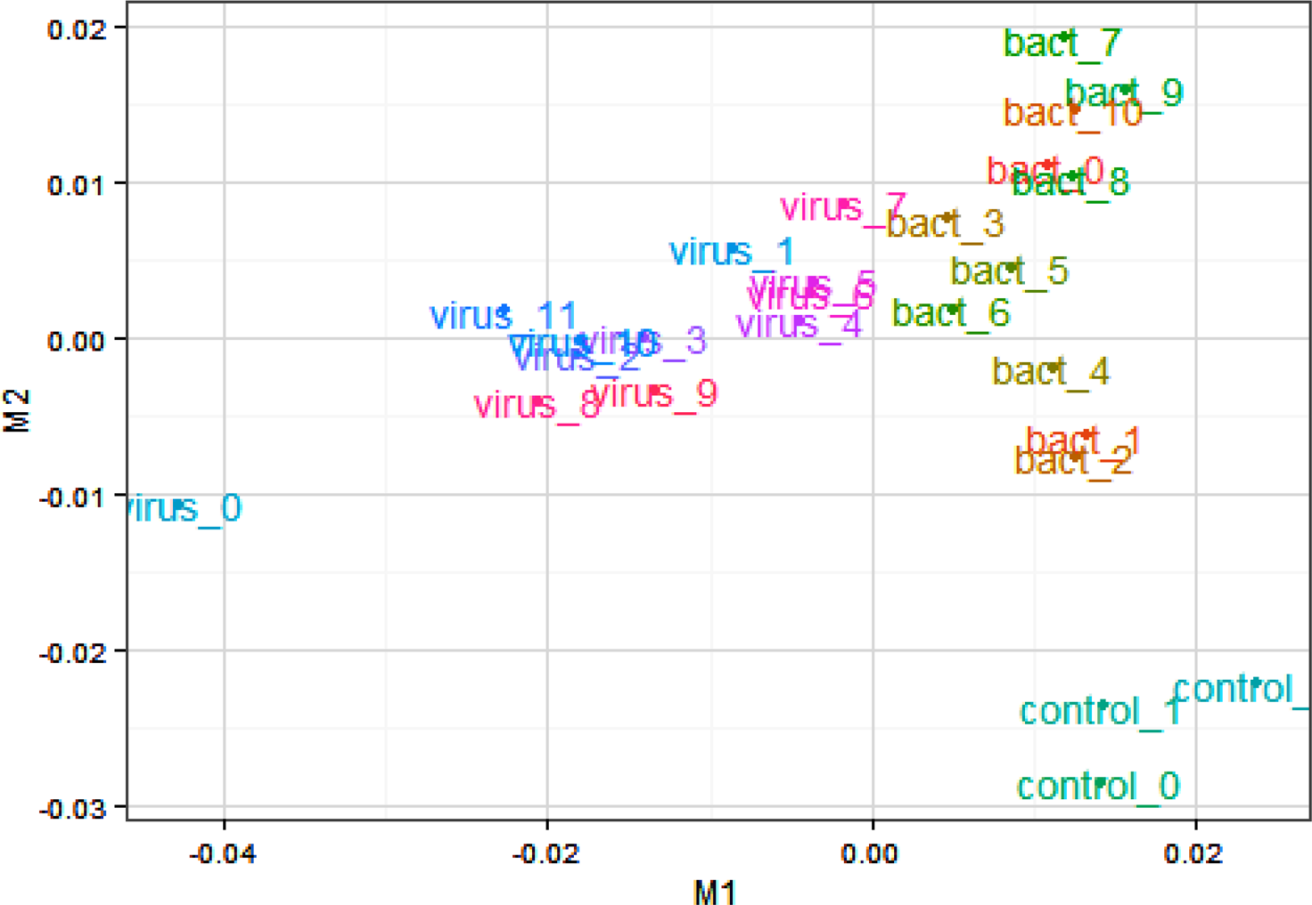
Multidimensional scaling plot of samples based on all genes. Legend: bact_0, bact_1 and bact_2 are *M*. *bovis* challenged; bact_3, bact_4, bact_5 and bact_6 are *P*. *multocida* challenged; bact_7, bact_8, bact_9 and bact_10 are *M*. *haemolytica* challenged; virus_0, virus_1, virus_2 and virus_3 are BRSV challenged; virus_4, virus_5, virus_6 and virus_7 are BVDV challenged and virus_8, virus_9, virus_10 and virus_11 are IBR challenged animals.

**Fig 2:**
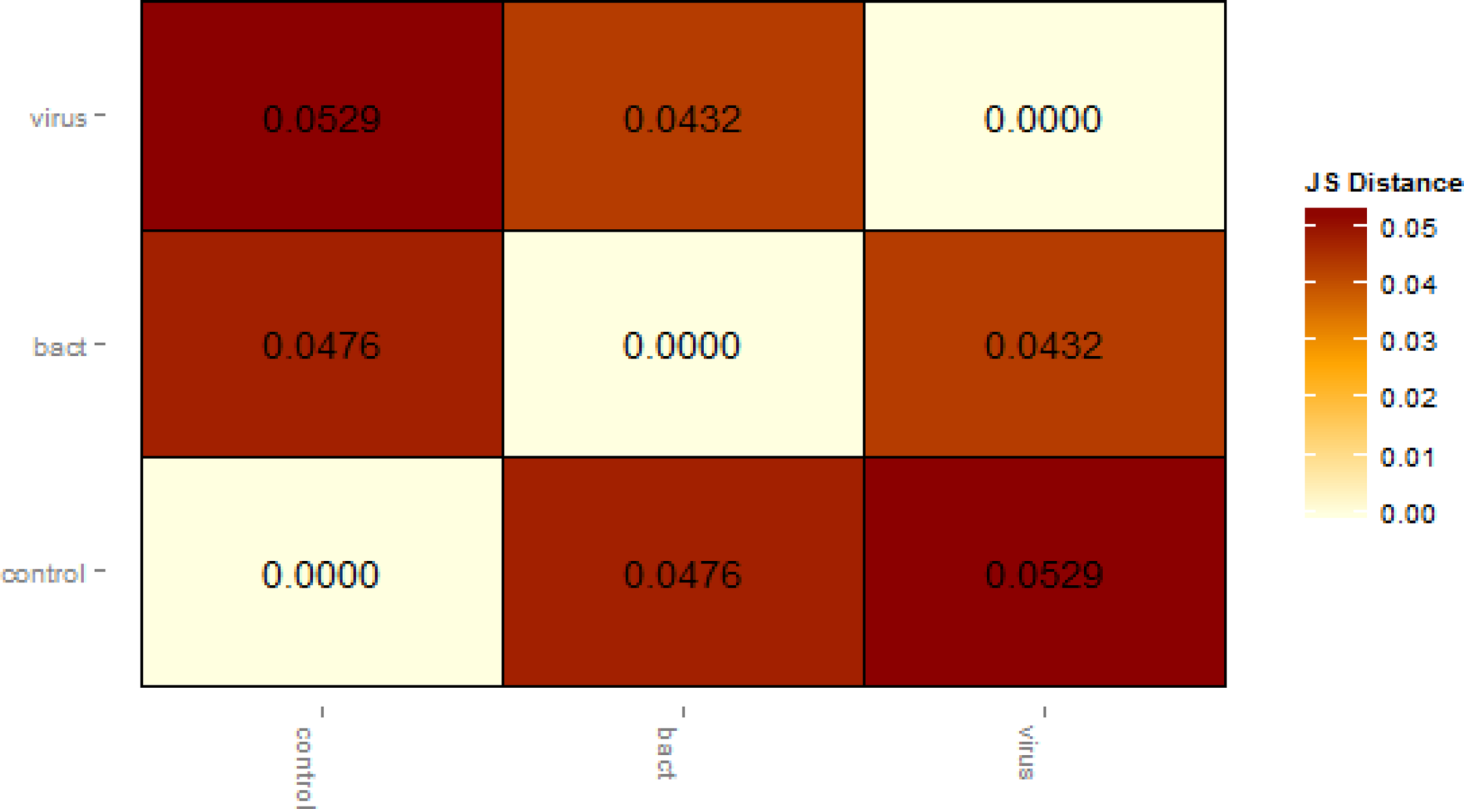
Heatmap showing the Jensen–Shannon (JS) divergence between challenge groups estimated from FPKM values for all genes.

**Fig 3:**
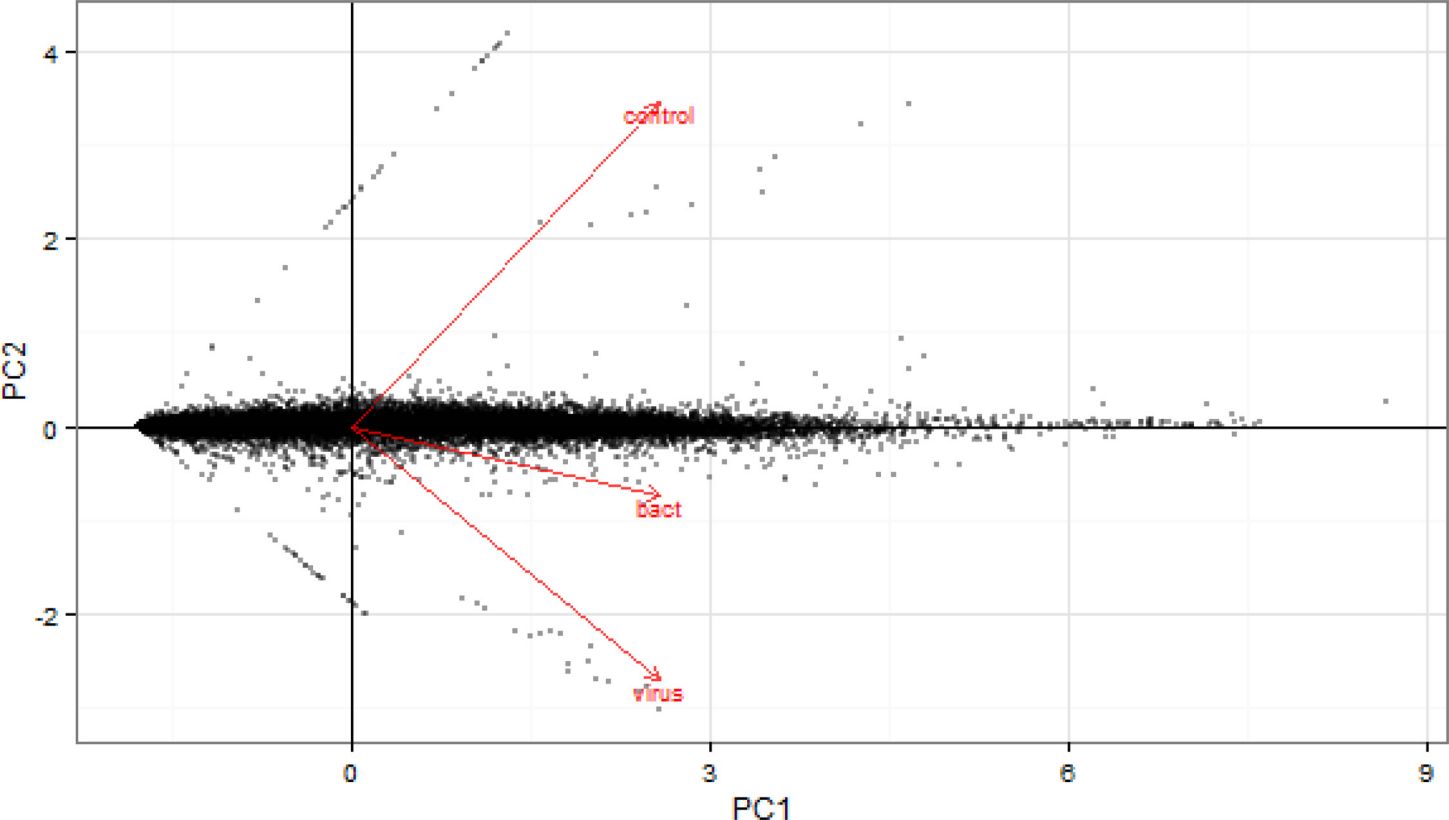
Principal component analysis for gene-level features.

**Fig 4:**
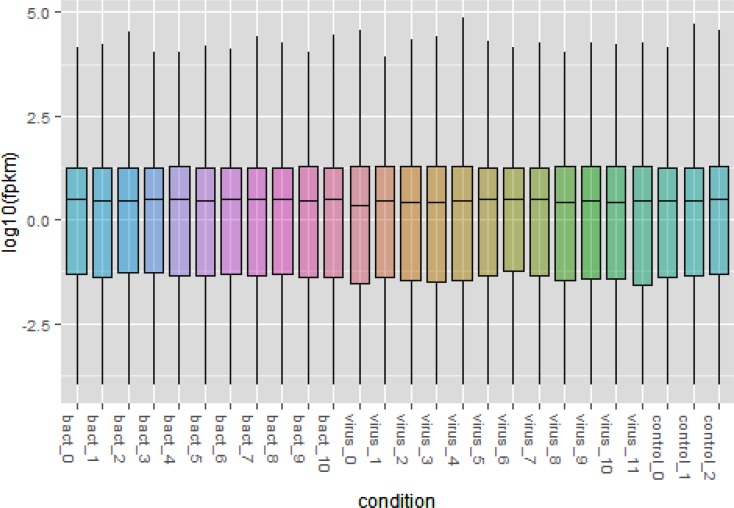
Dynamic range of FPKM values represented as log_10_ transformed FPKM values for each gene calculated for each biological replicate. Legend: bact_0, bact_1 and bact_2 are *M*. *bovis* challenged; bact_3, bact_4, bact_5 and bact_6 are *P*. *multocida* challenged; bact_7, bact_8, bact_9 and bact_10 are *M*. *haemolytica* challenged; virus_0, virus_1, virus_2 and virus_3 are BRSV challenged; virus_4, virus_5, virus_6 and virus_7 are BVDV challenged and virus_8, virus_9, virus_10 and virus_11 are IBR challenged.

To evaluate data quality, we first performed an analysis in which pairwise comparisons were made between all animals challenged with bacteria *vs* controls, all animals challenged with viruses *vs* control and all animals challenged with bacteria *vs* all animals challenged with viruses. The Cuffdiff output was also examined for reliability using several quality control methods. First, the variation between biological replicates was assessed using an MDS plot based on all expressed genes, which revealed a strong clustering of samples from control, bacterially and virally challenged animals with little variation between replicates (Fig 1). The first dimension clearly separated the virus- from the bacteria-challenged and control animals, while the second dimension separated controls from bacteria- and virus-challenged animals and suggests an ordering of pathogenicity. This postulate was also supported by the Jensen–Shannon divergence statistics between the challenged groups estimated from FPKM values for all genes (Fig 2) and by the principal component analysis (PC1 *vs* PC2; Fig 3), which show that the perturbation of gene expression was greatest for the animals challenged with viruses. The dynamic range of FPKM values was also evaluated by creating a boxplot of log_10_ transformed FPKM values for each biological replicate (Fig 4). The median FPKM values across the challenge groups were similar and the ranges indicate that our depth of sequence coverage was sufficient to allow the identification of genes with low transcript abundance.

### Differential expression and functional annotation

Differential expression analysis was performed separately for each pathogen challenge group in contrast to the control group to provide insights into pathogen-specific immune response. These analyses identified from 329 DE genes for the *M*. *bovis vs* control comparison to 4,123 for the IBR *vs* control comparison and the sign of the log_2_(fold change) was used to partition DE genes into up- and down-regulated groups (Table 2).

**Table 2:** Numbers of Up- and Down-Regulated Differentially Expressed Genes and Isoforms for each Challenge Group in Contrast to Controls.

	No. of Differentially Expressed Genes		
Comparison	Up-regulated	Down- regulated
BRSV *vs* control	1,543	1,942	
BVDV *vs* control	1,560	1,758	
IBR *vs* control	2,071	2,052	
*M*. *haemolytica vs* control	960	1,393	
*M*. *bovis vs* control	126	203	
*P*. *multocida vs* control	366	778	

The functional annotation analysis performed by DAVID for the DE genes found in the comparison of the BRSV challenged to control animals (S2 Table), revealed pathways related to brain diseases such as Parkinson’s, Huntington’s and Alzheimer’s. Additionally, pathways related to proteasome, oxidative phosphorylation, pyrimidine and purine metabolism, RNA-polymerase, spliceosome, cell adhesion molecules, EMC-receptor interaction, Wnt signaling, cytokine-cytokine receptor interaction and leukocyte transendothelial migration were identified as being enriched for DE genes along with other pathways with known roles in immune-response (S2 Table). The top five canonical pathways identified by IPA were oxidative phosphorylation (p≤8.97E-22), mitochondrial dysfunction (p≤4.15E-20), protein ubiquitination (p≤3.06E-11), role of macrophages, fibroblasts and endothelial cells in rheumatoid arthritis (p≤3.24E-09) and glucocorticoid receptor signaling (p≤2.33E-08). The upstream pathway regulators were predicted by IPA based upon the consistency of the direction of gene expression changes within the pathways enriched for DE genes (S3 Table). The most important upstream regulators predicted for the response to BRSV challenge included: lipopolysaccharide (LPS), tretinoin, *TNF*, *TGFB1* and beta-estradiol. With the exception of tretinoin, for which the activation state was not predicted; all other regulators were predicted to be activated in the challenged animals (and therefore inhibited in controls; S3 Table).

DAVID analysis of DE genes identified in the BVDV challenged *vs* control animals, revealed pathways for ECM-receptor interaction, focal adhesion, lysosome, complement and coagulation cascades, cell adhesion molecules, leukocyte transendothelial migration, cancer associated pathways, VEGF signaling, toll-like receptor signaling, TGF-beta signaling, MAPK signaling, and T cell receptor signaling (S4 Table). The IPA identified the hepatic fibrosis/hepatic stellate cell activation (p≤5.41E-17), acute phase response signaling (p≤3.14E-13), role of osteoblasts, osteoclasts and chondrocytes in rheumatoid arthritis (p≤1.36E-11), *IGF1* signaling (p≤5.83E-11) and leukocyte extravasation signaling (p≤2.47E-09) pathways. The five most significant upstream regulators were predicted to include *PPBP* (*TGB1*), *TNF*, *IRF6* (*LPS*), *TP53* and dexamethasone (S5 Table). While the activation status of *TNF* was not determined, all other regulators were activated in the challenged animals.

For the IBR challenged animals, DAVID analysis revealed similar pathways to those found for the BRSV and BVDV challenges, with a few exceptions (S6 Table). However, the IPA identified the role of macrophages, fibroblasts and endothelial cells in rheumatoid arthritis (p≤2.38E-11), role of osteoblasts, osteoblasts and chondrocytes in rheumatoid arthritis (p≤5.15E-10), B cell receptor signaling (p≤3.81E-09), acute phase response signaling (p≤4.65E-09) and PI3k signaling in B lymphocytes (p≤1.7E-08) as enriched for consistently regulated DE genes. The five most significant upstream regulators identified as controlling these processes were the same as those identified for the BVDV challenged animals (S7 Table).

Similar pathways to those found for the virus challenges were found for the animals challenged with the bacterial pathogens. In animals challenged with *M*. *haemolytica*, DAVID identified pathways enriched for DE genes including ECM-receptor interaction, focal adhesion, cell adhesion molecules, chemokine signaling, antigen processing and presentation, toll-like receptor signaling, primary immunodeficiency, T cell receptor signaling, natural killer cell mediated cytotoxicity and TGF-beta signaling (S8 Table). Pathways related to hepatic fibrosis/hepatic stellate cell activation (p≤1.28E-21), granulocyte adhesion and diapedesis (p≤1.65E-17), agranulocyte adhesion and diapedesis (p≤8.84E-15), type I diabetes mellitus signaling (p≤1.7E-08) and role of macrophages, fibroblasts and endothelial cells in rheumatoid arthritis (p≤4.12E-08) were identified by IPA. The most significant upstream regulators identified by IPA were essentially the same as reported for the virus challenges (S9 Table), however, *IFNG* and *IL4* regulators were also predicted. Only the activation statuses of *TGFB1* and *IFNG* could be predicted, which were activated and inhibited in challenged animals, respectively.

The DAVID and IPA results for the *M*. *bovis* (S10 Table) and *P*. *multocida* (S11 Table) challenges were similar to those for *M*. *haemolytica*. However, differences were found among the most significant of the upstream regulators (S12 and S13 Tables, respectively). For example, *IL1B* was identified for the M. *bovis* challenge. Furthermore, dexamethasone and beta-estradiol, previously identified as being among the most important upstream regulators for the BVDV and BRSV challenges, were also found to be important regulators for response to *P*. *multocida* challenge. In general, the activation statuses of these regulators could not be determined; however, *TGFB1* and dexamethasone were predicted to be activated in *P*. *multocida* challenged animals.

**Fig 5:**
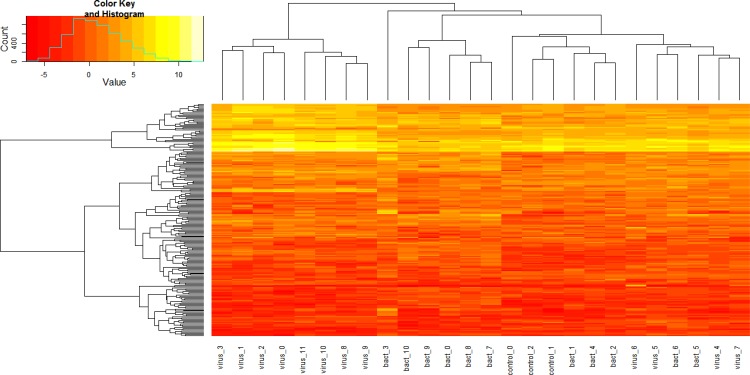
Heatmap for 200 differentially expressed genes with the greatest fold differences from all pathogen challenges using hierarchical clustering analysis. Hierarchical clustering of gene expression profiles in all samples. Each row represents a gene and each column an animal. The extent of expression of each gene in each sample is indicated by a color code. The color key ranges from saturated red for log_**2**_ ratios less or equal to -5.0 to saturated yellow for log_**2**_ ratios greater than or equal to 10. Red indicates an increased gene expression in the challenged animals. Legend: bact_0, bact_1 and bact_2 are *M*. *bovis* challenged; bact_3, bact_4, bact_5 and bact_6 are *P*. *multocida* challenged; bact_7, bact_8, bact_9 and bact_10 are *M*. *haemolytica* challenged; virus_0, virus_1, virus_2 and virus_3 are BRSV challenged; virus_4, virus_5, virus_6 and virus_7 are BVDV challenged and virus_8, virus_9, virus_10 and virus_11 are IBR challenged.

We found 5,757 unique genes to be DE relative to the controls across all pathogen challenge groups. From this list of genes, we identified the 200 DE genes with the greatest variance in FPKM values across all biological replicates and used them to perform a hierarchical clustering analysis based upon gene expression (Fig 5). This analysis was able to correctly cluster the biological replicates for BRSV, IBR, and the controls, while clusters for IBR and *M*. *haemolytica* each contained a member from a different challenge pathogen and replicates for *M*. *bovis* and *P*. *multocida* were not well clustered. This is consistent with the finding that these challenge groups had the fewest DE genes (Table 2) since the strategy for gene sampling primarily selected genes with large expression variances which tended to be drawn from the challenge groups with the largest number of DE genes (Table 3). Of the 200 genes, 25 were DE in all challenge groups (Table 3), indicating that these genes are predictive of infection regardless of specific etiology. However, several among these 200 genes were exclusive to a single pathogen challenge, and these genes are of significant biological interest for understanding the bovine pathogen-specific immune response, and are likely to be useful for predicting pathogen-specific infections among cattle within various production environments.

**Table 3:** Differentially Expressed Genes with the Largest Expression Variances by Challenge Group.

Pathogen Challenge	Genes
All pathogens	*ACTG2*, *CTHRC1*, *CXCL9*, *GP2*, *HERC6*, *IFI44*, *IFIT1*, *IFIT3*, *ISG15*, *MMP9*, *OAS1*, *PGLYRP1*, *PI16*, *RAPGEF5*, *RSAD2*, *S100A8*, *S100A9*, *SFRP2*, *SFRP4*, *SLC6A15*, *TF*, *TGM3*, *ULBP3*, *VCAN* and *ZBP1*
At least two viral pathogens	*FOSB*, *GFRA2*, *GZMB*, *NUPR1*, *WSCD2*, *AIRE*, *BPI*, *CDHR1*, *CHODL*, *CLEC10A*, *EDIL3*, *ESR2*, *IFITM3* and *MCHR1*
At least two bacterial pathogens	*CDH10*, *DGAT2*, *MMP13*, *PAMR1*, *PLAC8*, *PLIN4*, *PRSS35*, *MGAM*, *MGAT3*, *ADC*, *FABP7*, *MAL2*, *OLFM4* and *SEMA3C*
BRSV	*FRZB*, *MT1E*, *NPTX1*, *TSPAN18* and *CA4*
BVDV	*ALB*, *ITIH2*, *KRT24*, *MMP7* and *TTR*
IBR	*AHNAK*, *ASZ1*, *BST2*, *CD1B*, *KCNE1L*, *LILRA4*, *PLCXD3*, *RNF24* and *TMEM213*
*M*. *haemolytica*	*BMPR1B*, *COL6A6*, *KIR3DL2*, *LYNX1*, *MAD2*, *PF4*, *PRND*, *SOSTDC1*, *TDRD1* and *TRIL*
*M*. *bovis*	*EBD* and *TSPAN1*
*P*. *multocida*	*ACOX2*, *ACSM1*, *BTNL9*, *CYP1A1*, *FABP4*, *GSTT3*, *SIX1*, *TECTB* and *TUSC5*

**Fig 6:**
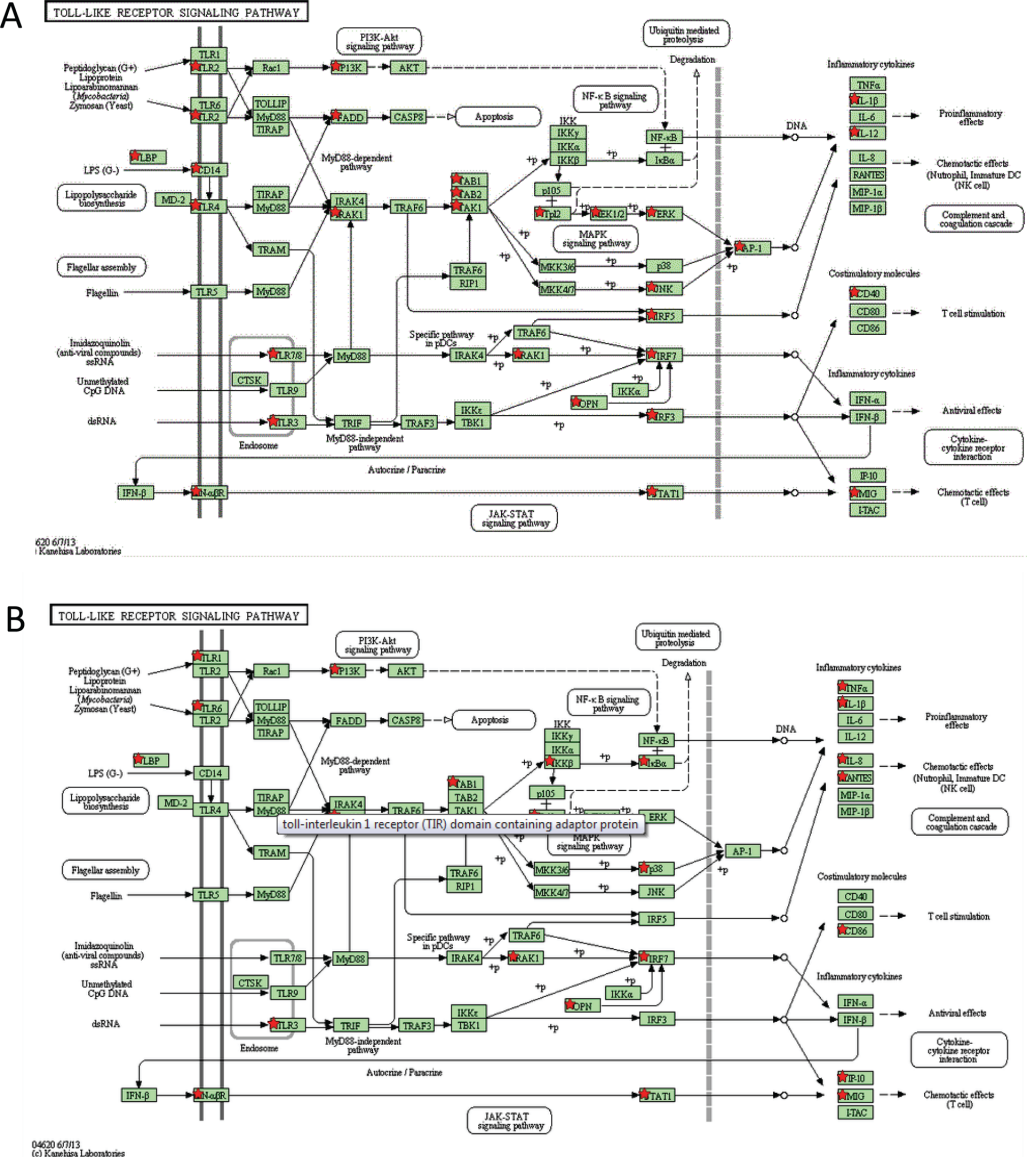
Differentially expressed genes enriched within the toll-like receptor pathway. **A**. IBR challenged animals. **B**. *M*. *haemolytica* challenged animals. Red stars indicate the differentially expressed genes in each pathway.

While some pathways enriched for DE were shared among several of the pathogen challenges, different genes appeared to be responsible for their regulation. For example, pattern-recognition receptors may be pathogen-specific. To examine this, we compared the DE genes enriched within the toll-like receptor pathway in both the IBR and *M*. *haemolytica* challenges. Although some genes were in common, the activation or inactivation of signaling pathways appears to be caused by the regulatory mechanisms of specific downstream transcription factors (Fig 6).

Recently, a GWAS has reported 116 genomic regions associated with BRDC susceptibility in Holstein calves which had been naturally exposed to the pathogens responsible for disease [23]. The 1 Mb regions flanking each QTL harbor 649 annotated candidate genes, of which, 142 were found to be DE in our analysis, with 27 identified as potential upstream regulators likely responsible for the transcriptional changes detected in pathways enriched for DE genes (S14 Table). Some of the QTL regions contain at least six genes that were DE in response to multiple challenge pathogens and at least one of the genes was predicted to be an upstream regulator. In particular, the QTL regions from 107–108 Mb on BTA3, 67–68 Mb on BTA13, 30–31 Mb on BTA15, 72–73 Mb on BTA17 and 55–56 Mb on BTA18 harbor from 6 to 16 genes that were DE in animals challenged by all pathogens except for *M*. *bovis*. The QTL region from 65–66 Mb on BTA18 harbors 10 DE genes for which the upstream predicted regulators BCL2-Associated X Protein (*BAX*) and Protein Phosphatase 1, Regulatory Subunit 15A (*PPP1R15A*) were located in the syntenic QTL region at 55–56 Mb. The 10 genes in this QTL region were found to only be DE in response to challenge by the three viral pathogens.

## Discussion

Efforts have been made to decrease the use of antibiotics in livestock production and to find alternative methods to control disease. Global gene expression analyses have been used to identify prognostic signatures in humans, i.e., for the discovery of gene sets that may predict patient outcomes relative to treatment for a specific cancer [24,25]. BRDC is caused by the interaction of stressors which are often temporally coincident with natural exposures to viral and bacterial agents which collectively lead to the manifestation of clinical signs and lung pathology. However, each pathogen may trigger a specific immune response. The RNA sequencing approach adopted in this study provides an expression pattern of genes that may be helpful for predicting whether a pathogen-specific immune response mechanism is activated and for the development of new therapeutic treatment options (i.e., drugable targets).

Since BRD is multifactorial and can be caused by a number of pathogens individually or as co-infections [26], determination of disease etiology can be difficult. We found both common and pathogen-specific differences in the bronchial lymph node transcriptome profiles of animals challenged with viruses and bacteria known to be common causes of BRD. The DE genes and pathways that were found to be common to all pathogen challenges were up-regulated in the challenged animals and appear to primarily be related to the innate immune response, which enables a rapid antimicrobial host defense prior to the development of the adaptive immune response. This was consistent with the sampling of steers for the challenge experiment which had either no, or low, blood titers against the challenge pathogens. However, the innate immune system may also be involved in determining which antigens the acquired immune system responds to and the manner of that response [27].

We identified several pathways which underlie the immune response mechanisms that are induced following infection. The innate immune system, which responds to all pathogens, recognizes pathogen-associated molecular patterns (PAMPs) by host pattern-recognition receptors (PRRs). The list of PRRs which are known to sense PAMPs is extensive, and includes members of at least four gene families: Toll-like receptors (TLRs), nucleotide oligomerization domain like receptors, C-type lectin receptors and retinoic acid-inducible gene I like receptors. PRR ligation stimulates several signaling pathways that result in the activation of nuclear factor-**κ**B, mitogen-activated protein kinases (MAPKs) and interferon regulatory factors, which mediate the production of cytokines and type I interferons needed for an effective innate response [28–32]. The pro-inflammatory state that results from these mechanisms is necessary for the generation of a robust antimicrobial environment and for the appropriate stimulation of the adaptive immune response [32].

The common pathways underlying response to two or more of the challenge organisms include pathways with major roles in innate immunity that are less pathogen-specific. For example, we found the toll-like receptor pathway and MAPK signaling which are responsible for the phosphorylation and activation of kinases which regulate cytokine production [33], TGF-beta signaling, which functions in cytokine regulation [34] and natural killer cell mediated cytotoxicity whereby populations of lymphocytes are activated to cytotoxicity via the production of cytokines and chemokines [35]. The cytokine and chemokine-related pathways were all up-regulated in the challenged animals. Cytokines and chemokines have important functions in the innate and adaptive immune systems. The production of cytokines by innate immune cells occurs as an acute response to inflammation and infection across diverse etiologies. The release of cytokines and chemokines orchestrates immune responses through communication with other cells. For the immune response to efficiently function, the synthesis and release of cytokines must be controlled [36] and the pathways enriched for DE genes detected in our analyses of these data are involved in this process. It is important to note that cytokines underlying the control of viral immunity differ from those that are most relevant to anti-bacterial defense. For example, an effective immune response to BRSV, IBR and BVDV requires the development of cytotoxic T cells, which are critical for the elimination of virus infected cells. Production of interleukin 12 and interferon gamma by T helper type 1 cells facilitates the priming of CD8 cytotoxic T cells. Whereas, in humoral immunity, antibody production is associated with interleukin 4 expression and interleukin 17 stimulates a neutrophil response in the defense against many bacterial pathogens [37].

Despite the finding of common pathways enriched for DE genes across challenge pathogens, different genes appear to be responsible for pathway activation. For example, the DE genes that are enriched in the toll-like receptor pathway differ for the IBR challenged and *M*. *haemolytica* challenged groups (Fig 6). In response to the *M*. *haemolytica* challenge, *TLR1* and *TLR6* are induced while for IBR, *TLR2* and *TLR4* are induced. TLR2 and TLR4 proteins are present on the surface of immune cells and are known to be involved in the recognition of several viruses via the detection of envelope glycoproteins [38]. The activation of TLRs initiates downstream signaling cascades which regulate intracellular kinases and may stimulate an inflammatory and an antigen-specific immune response. The signaling pathways associated with each TLR differ and may involve different biological responses [39]. Our results suggest that *TLR1* and *TLR6* mediate the innate immune response to *M*. *haemolytica* while *TLR2* and *TLR4* mediate response to infection by IBR and other viruses.

We also detected evidence of adaptive immune response mechanisms, in particular the enrichment of DE genes within the B and T cell receptor signaling pathways. Lymphocytes can recognize a large number of different antigens including bacteria, viruses, and other disease-causing organisms [37]. While B cells are critical for the humoral immune response, T helper cells are important for initiation of the humoral response to T dependent antigens. T lymphocytes are also involved in cell-mediated immune responses, both as T helper type 1 cells and as cytotoxic T cells. B cell-related pathways were not detected in response to challenge by any of the bacterial agents which is puzzling because the development of an antibody response usually occurs for infections by each of the bacterial pathogens. Presumably these were induced early in the response to challenge and had abated by the time the animals were euthanized. However, the IPA results revealed that pathways related to B cell receptors were among the most important in the animals’ responses to virus challenge.

In addition to the B cell receptor signaling pathway, which was induced only for the virus challenges, pathways including the regulation of actin cytoskeleton, ErbB signaling, RNA polymerase and degradation, VEGF signaling and oxidative phosphorylation related to mitochondrial activity were also important for response to viral infection. Some of these pathways have clear roles in immune response, e.g., regulation of the actin cytoskeleton has a crucial role in important cellular processes required for normal immune function, including locomotion, intercellular interactions, endocytosis, signal transduction and maintenance of cell morphology [40] and oxidative phosphorylation which is required for memory T cell metabolism [41]. We also found pathways related to brain diseases [42] in the IPA of the DE genes in animals challenged with BRSV which have a key role in mammalian immune response through the regulation of apoptosis during infection. In the DAVID analysis of this gene set, the most enriched functional clusters were mitochondrial and oxidative phosphorylation (S2 Table). Mitochondria have recently been recognized as having an important role in the innate immune response against viral pathogens [32].

Neurodegenerative disease-related pathways including Huntington’s, Parkinson’s and Alzheimer’s diseases were induced in the animals challenged with BRSV and IBR. There are many shared genes between these 3 pathways that relate to neurological disease. For example, all three include the activation of *COX5A* and *COX5B* which are cytochrome C oxidase subunit enzymes in the mitochondrial respiratory chain and defects in mitochondrial function are associated with all three diseases. However, activation of these genes by BRSV and IBR is not likely to be associated with neurological disease in infected cattle. Rather, our findings suggest that neurologic interference may be a common manifestation of infection by pathogenic viruses. A recent study found that neurodegenerative disease-related pathways were induced in HIV-infected peripheral blood mononuclear cells [43]. Potential upstream regulators of these pathways and their target molecules were identified in our analyses (S3, S5, S7, S9, S12 and S13 Tables) and this information may be useful for the development of novel therapeutics. IPA upstream regulator analysis attempts to identify the cascade of upstream transcriptional regulators that are responsible for the observed gene expression changes [44] and whether they are likely activated or inhibited.

LPS was identified as an upstream regulator in the response to challenge by all pathogens and agents leading to LPS stimulation may provide new therapy options for the prevention of respiratory diseases in cattle. LPS treatment is known to activate the innate immune response and induce interferon-stimulated genes and proinflammatory cytokines in hepatocytes, inhibiting, for example, hepatitis B virus replication [45]. Our results indicate that LPS also seems to induce a cascade of antiviral genes [46] in addition to antibacterial genes. The LPS activated immune response to bacterial infection results in the production of NKT cells and IFN**γ** [47]. Additionally, TNF, which was also identified as an upstream regulator of gene expression response to all challenge pathogens may also deserve consideration in the development of treatment options which regulate immune cells. Anti-TNF is currently being used to treat human inflammatory and autoimmune diseases, for example, rheumatoid arthritis. Use of this drug has been associated with decreased immune responses to a variety of pathogens [48,49].

Twenty five of the 5,757 DE genes were common to all pathogen challenges (Table 3) and function in clusters of secreted and extracellular proteins such as the ubiquitin-like modifier *ISG15*, which is induced by IFN-**α**/**β** and plays an essential role in innate immunity against viral and bacterial infections [50], *Tf* which is important to cell proliferation through the supply of iron for DNA synthesis [51], *MMP9* which is an important protease that is involved in the breakdown of the extracellular matrix and leukocyte recruitment [52] and *CXCL9* which is involved in T cell trafficking [53]. Further, the myeloid-related protein encoded by *S100A9* which is a calcium and zinc binding protein has a role in the inflammatory and immune response and is up-regulated in peripheral blood mononuclear cells of severe acute respiratory syndrome patients [54]. It can enhance neutrophil adhesion, chemotaxis, and bactericidal activity and may be an interesting target for genetic selection.

Hierarchical clustering using FPKM values for all biological replicates for the 200 DE genes with the largest expression variances across biological replicates was nearly capable of correctly clustering all replicates into their respective challenge groups (Fig 5). *P*. *multocida* was an exception and the challenge with this pathogen was not ideal, since *M*. *bovis* was also isolated from the lungs of these challenged animals. Despite this, we have identified bronchial lymph node gene expression signatures which characterize the immune response and are predictive of the pathogens responsible for BRDC in cattle.

The difficulty in achieving a single infection with any of the BRDC pathogens was underscored in this study by our use of commercial animals. Cultures of the upper respiratory tract from the steers revealed the expected common bacterial pathogens. However, culture of lungs from these steers predominantly demonstrated the pathogen that was introduced by experimental challenge. Our selection of the bronchial lymph node for RNA sequencing was based upon the fact that lymphocytes within the node drain the lung and therefore their RNA expression represents a response to pathogens in the lung [55]. Thus, lymphocyte responses to bacterial agents found in the upper respiratory tract, where they normally occur as commensals, were not expected to be observed in transcripts from the bronchial lymph nodes.

Many DE expressed genes are located in previously identified QTL regions associated with risk of BRD in Holstein calves [23]. Among these are several key upstream regulators which are predicted to regulate the expression of numerous downstream genes that were DE between challenged and control animals. Polymorphisms within regulatory elements controlling the expression of these genes and their isoforms or within the primary transcripts themselves are strong candidates for the detected QTL. Due to the difference in breeds between this and the GWAS [23] and because animals in this study were randomly assigned to challenge groups without regard to their BRD susceptibility, the transcript variants for the DE genes reported in S14 Table do not inherently provide strong candidates for the causal variants underlying the QTL. Rather, variant detection using genomic DNA from cases and controls drawn from the GWAS population offers the greatest likelihood of identifying the causal variants.

## Summary and Conclusions

The majority of DE genes discovered in this study were up-regulated in the challenged animals and involved with innate immunity regardless of disease etiology. Among these genes were those coding for pattern-recognition receptors which appear to stimulate the same metabolic pathways. A set of the 200 most variable of the DE genes was able to partially classify each replicate into its respective challenge group and may be useful for predicting the immunopathogenesis of disease caused by each pathogen and the effects that genetic manipulation might have on disease outcomes. Key upstream regulators were identified which provide possible targets for the development of novel molecular therapies and studies exploring the effects of manipulating their signaling on the modulation of bovine immune function should be considered. One hundred and forty two DE genes, some of which are key upstream regulators were found to be located in previously identified QTL regions associated with risk of BRD. Our analyses of these data provide new insights and perspectives into the genetic basis of BRDC susceptibility and the molecular mechanisms underlying bovine immune responses to respiratory disease which may be useful for the identification of large effect loci underlying differential susceptibility and for the development of new therapeutic intervention strategies.

## Supporting Information

S1 Table Sample identification for RNA-Seq data corresponding to each of the experimentally challenged animals within the NCBI Sequence Read Archives. (XLSX)

S2 Table List of differentially expressed genes found for the BRSV *vs* control comparison and enriched GO terms from the DAVID analyses. Tabs are identified according to the type of DAVID analysis performed.
(XLSX)

S3 Table Upstream regulators identified by Ingenuity Pathway Analysis for genes found to be differentially expressed between BRSV and controls. Activation status refers to the Control group.
(XLS)

S4 Table List of differentially expressed genes found for the BVDV *vs* control comparison and enriched GO terms from the DAVID analyses. Tabs are identified according to the type of DAVID analysis performed.
(XLSX)

S5 Table Upstream regulators identified by Ingenuity Pathway Analysis for genes found to be differentially expressed between BVDV and controls. Activation status refers to the Control group.
(XLS)

S6 Table List of differentially expressed genes found for the IBR *vs* control comparison and enriched GO terms from the DAVID analyses. Tabs are identified according to the type of DAVID analysis performed.
(XLSX)

S7 Table Upstream regulators identified by Ingenuity Pathway Analysis for genes found to be differentially expressed between IBR and controls. Activation status refers to the Control group.
(XLS)

S8 Table List of differentially expressed genes found for the *M*. *haemolytica vs* control comparison and enriched GO terms from the DAVID analyses. Tabs are identified according to the type of DAVID analysis performed.
(XLSX)

S9 Table Upstream regulators identified by Ingenuity Pathway Analysis for genes found to be differentially expressed between *M*. *haemolytica* and controls. Activation status refers to the Control group.
(XLS)

S10 Table List of differentially expressed genes found for the *M*. *bovis vs* control comparison and enriched GO terms from the DAVID analyses. Tabs are identified according to the type of DAVID analysis performed.
(XLSX)

S11 Table List of differentially expressed genes found for the *P*. *multocida vs* control comparison and enriched GO terms from the DAVID analyses. Tabs are identified according to the type of DAVID analysis performed.
(XLSX)

S12 Table Upstream regulators identified by Ingenuity Pathway Analysis for genes found to be differentially expressed between *M*. *bovis* and controls. Activation status refers to the Control group.
(XLS)

S13 Table Upstream regulators identified by Ingenuity Pathway Analysis for genes found to be differentially expressed between *P*. *multocida* and controls. Activation status refers to the Control group.
(XLS)

S14 Table Differentially expressed genes located within genomic regions previously shown by GWAS to harbor QTL associated with susceptibility to BRDC. (XLSX)
